# Two-Step Separation of Chitin from Shrimp Shells Using Citric Acid and Deep Eutectic Solvents with the Assistance of Microwave

**DOI:** 10.3390/polym11030409

**Published:** 2019-03-03

**Authors:** Dandan Zhao, Wen-Can Huang, Na Guo, Shuye Zhang, Changhu Xue, Xiangzhao Mao

**Affiliations:** 1 College of Food Science and Engineering, Ocean University of China, Qingdao 266003, China; Lizzy_Dan@163.com (D.Z.); hwc@ouc.edu.cn (W.-C.H.); gnaever@163.com (N.G.); xuech@ouc.edu.cn (C.X.); 2 State Key Laboratory of Advanced Welding and Joining, Harbin Institute of Technology, Harbin 150001, China; syzhang@hit.edu.cn; 3 Laboratory for Marine Drugs and Bioproducts, Qingdao National Laboratory for Marine Science and Technology, Qingdao 266237, China

**Keywords:** chitin, shrimp shells, deep eutectic solvents, citric acids

## Abstract

In this research, a two-step extraction approach was developed for chitin preparation from shrimp shells by utilizing citric acids and deep eutectic solvents (DESs), which effectively removed minerals and proteins. In the first step, minerals of shrimp shells were removed by citric acid, and the demineralization efficiency reached more than 98%. In the second step, the removal of protein was carried out using deep eutectic solvents with the assistance of microwave, and the deproteinization efficiency was more than 88%. The results of scanning electron microscopy (SEM), Fourier transform infrared (FT-IR) spectroscopy, X-ray diffraction analysis (XRD), and thermogravimetric analysis (TGA) showed that the quality of DES-prepared chitin was comparable to that of traditional acid/alkali-prepared chitin. These results were realized without utilizing hazardous chemicals, which are detrimental to the environment. This research indicates that a DES-based preparation approach has the potential for application in the recovery of biopolymers from natural resources.

## 1. Introduction

Chitin is considered the second most plentiful biopolymer in nature after cellulose [1]. It is a linear amino polysaccharide comprised of β-(1-4)-connected 2-deoxy-2-acetamido-D-glucose units [2]. The main source of chitin is crustacean shells derived from shrimp and crab [3]. Chitin is considered an important material because of its various characteristics, such as biodegradability, biocompatibility, non-toxicity, low immunogenicity, and thermal stability [4,5]. Therefore, chitin and its derivatives are widely used in cosmetic [6], food [7], agricultural [8], tissue engineering [9], waste water treatment [10], and packaging material applications [11]. It is reported that shrimp accounts for about 45% of processed seafood [12]. The shrimp processing industry produces massive amounts of by-products, such as shrimp shells. These shrimp by-products are usually applied for low-value animal feeds and biological fertilizers [13]. Therefore, it is particularly critical to convert by-products into high-value products. Shrimp shells are mainly composed of chitin, proteins, and minerals [14]. Chitin is extracted from shrimp shells by demineralization and deproteinization procedures.

In current industrial processing, methods for chitin preparation include chemical treatments, enzymatic reactions, and microbial fermentation [1,15,16]. Conventional chemical extraction of chitin requires strong acids and alkali to eliminate minerals and proteins [17]. However, using these harsh chemicals is detrimental to the environment. In addition, although enzymatic reactions and microbial fermentation methods avoid this drawback, the incomplete elimination of minerals and proteins limits the application of these methods [14]. 

Recently, chitin extraction by ionic liquids (ILs) from crustacean shells has been reported as an alternative method [14]. However, the main disadvantages of ILs are their high cost, toxicity, and complicated synthesis steps, which limit their application [18]. Deep eutectic solvents (DESs) are recognized as novel ionic liquid analogues [19]. DESs are composed of a hydrogen bond donor (HBD) and a hydrogen bond acceptor (HBA) that are capable of self-association by interactions with special hydrogen bonds to constitute a eutectic mixture with a lower melting point than those of every single ingredient [20,21]. Compared to ILs, DESs show similar physico-chemical characteristics while they are more advantageous because of their low price, non-toxicity, low flammability, and biodegradability [19,22]. Therefore, DESs are widely used in various applications, such as dissolution and extraction procedures, catalysis, organic synthesis, metal processing, electrochemistry, and analytical chemistry [19,21]. Moreover, studies have reported that DESs can also be used to dissolve and extract materials from natural products, such as phenolic compounds, biodiesel, starch, lignins, cellulose, and other carbohydrates [21,23].

In this work, the DESs composed of mixtures of betaine hydrochloride (betaine HCl)-urea, choline chloride (ChCl)-urea, ChCl-ethylene glycol, and ChCl-glycerol were exploited to produce chitin with the assistance of microwave irradiation. To the best of our knowledge, the four DES systems have been widely used in various studies but there has been no attempt to obtain chitin from shrimp shells. Furthermore, the obtained chitin was examined by FT-IR, XRD, TGA, and SEM, and the reusability of DESs was also assessed.

## 2. Materials and Methods

### 2.1. Materials

The shrimp shells were dried in the oven at 90 °C and pulverized into powder with a particle size of 0.355 nm by using a grinder. Choline chloride were acquired from Yuanye Bio-Technology (Shanghai, China). Urea and coomassie brilliant blue G-250 were purchased from Solarbio (Shanghai, China). Betaine hydrochloride was purchased from Macklin (Shanghai, China). Ethylene glycol, glycerol, citric acid, hydrochloric acid, sodium hydroxide, lithium chloride (LiCl) and *N*,*N*-dimethylacetamide (DMAc) were acquired from Sinopharm Chemical Reagent Co., Ltd. (Shanghai, China).

### 2.2. Synthesis of DESs

**Figure 1:**
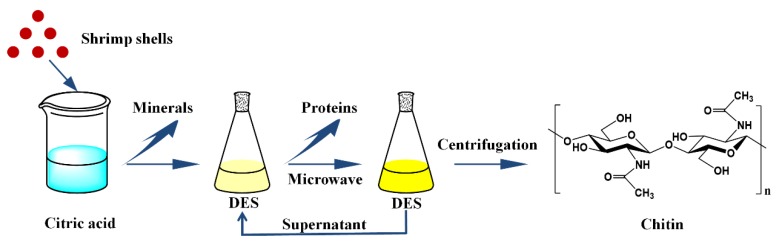
Schematic depiction of two-step separation of chitin from shrimp shells.

In a typical process, the hydrogen bond donor (HBD) and acceptor (HBA) ingredients were mixed and heated at a certain temperature with magnetic stirring at an optimal ratio until homogenous and transparent solutions were obtained. Details of the preparation of the DESs are shown in Table 1. DESs used in this study are presented in Figure 1.

### 2.3. Preparation of Chitin

Figure 1 shows the schematic depiction of two-step separation of chitin from shrimp shells. Chitin preparation by utilizing citric acids and DESs was conducted as follows. The shrimp shells were treated with 10% citric acid for demineralization. The pretreated samples were dispersed in DESs with different shrimp shell/DES ratios of 1:5, 1:10, 1:15, and 1:20. Then, the mixtures were heated by microwave irradiation at various times (1 min, 3 min, 5 min, 7 min and 9 min). To avoid excessive heating of the mixtures, 2 to 3 s pulses were used, and the mixtures were stirred manually with a glass rod to ensure uniform dispersion of the shrimp shell powders in the DESs. Next, chitin and the DESs were separated via centrifugation. The chitin was gathered and rinsed with distilled water. The supernatants were collected for next use. After separation, the chitin was dried in an oven at 80 °C. The chitin yield was evaluated by calculating the ratio of the weight of extracted chitin to raw shrimp shells [24]. The DESs were used for five cycles without purification, and the recyclability of the DESs was assessed. 

The mineral content was measured by the combustion of the sample (1–2 g) in a muffle furnace (525 °C) to a constant weight [25]. The demineralization (DM) efficiency was calculated according to the Equation (1):

DM (%) = [(M_1_ − M_2_)/M_1_] × 100% (1)

where M_1_ and M_2_ represent the mineral content of the crude shrimp shells and prepared chitin, respectively.

The protein content of the samples was evaluated through the Bradford method [26]. The percentage of deproteinization (DP) was calculated according to the Equation (2):

DP (%) = [(P_1_ − P_2_)/P_1_] × 100% (2)

where P_1_ and P_2_ represent the protein content of the crude shrimp shells and prepared chitin, respectively. All the above experiments were performed in triplicate. 

For comparison with the DES-extracted chitin, acid/alkali extraction was conducted [7]. Demineralization was performed by adding 5% (*w*/*v*) HCl solution to the shrimp shells (30 g). The mixtures were stirred for 1 h at ambient temperature. Afterwards the reacted samples were rinsed with distilled water and gathered by centrifugation. Next, deproteinization was performed by adding 10% (*w*/*v*) NaOH to the samples, and the mixtures were stirred for 2 h at 95 °C. The resulting samples were rinsed with distilled water until a neutral pH was achieved, and they were dried in an oven at 80 °C.

### 2.4. Characterization

Chitin acetylation was determined by acid/alkali titration [27]. Dried sample of 0.2 g was dispersed in 30 mL of 0.1M HCl solution and stirred for 1 h. Then, the mixture was titrated with 0.1 M NaOH solution. The degree of acetylation (DA) of chitin was expressed by the following equation:

(3) $$\mathrm{DA}\;\left(\%\right)\;=\;[1－\frac{\left(C_{{}^{1}}V_{1}-C_{0}V_{0}\right)\times 0.016}{m\times (1-W)\times 0.0994}]\times 100\%$$

where *C*_1_ and *C*_0_ are the concentrations of HCl and NaOH solution in mol/L, respectively. *V*_1_ and *V*_0_ are the consumption volumes of HCl and NaOH solution in ml, respectively. m is weight of the sample in g. W is moisture content of the sample in %. 0.016 is molecular weight of NH_2_ in 1 mL 0.1 M HCl solution in g. 0.0994 is the theoretical NH_2_ content. The above experiment was repeated three times.

The molecular weight (Mw) of prepared chitin was measured by a Ubbelohde viscometer (Shenbo Glass Instrument Co., Ltd., Shanghai, China) at 30 °C. Chitin was dissolved in 5% (*w*/*w*) LiCl/DMAc and prepared into different concentrations of chitin solutions (0.03–0.05 g/dL). The Mw was calculated using the Mark–Houwink–Sakurada equation [28].

[ŋ] = KM_w_^α^ (4)

where [ŋ] represent intrinsic viscosity, K = 7.6 × 10^−5^ dL/g, α = 0.95.

The surface morphologies of the samples were examined through utilizing a JEM-1200EX scanning electron microscope (SEM, JEOL, Tokyo, Japan). The samples were freeze-dried and then were covered with a platinum film (Pt coating) and linked to a metal stub before the detection. The samples were observed at an acceleration voltage of 10 kV.

The Fourier transform infrared spectra (FT-IR) of the samples were collected with a Nicolet iS10 spectrometer (Thermo Fisher Scientific, Massachusetts, USA) over the wavenumber between 4000 and 500 cm^−1.^

The X-ray diffraction patterns (XRD) were conducted on a MinFlex 600 diffractometer (Bruker, Leipzig, Germany) using Cu Kα radiation (λ = 1.54056 nm) at 40 kV. The diffraction data were collected with a 2θ angle in the scope of 5° to 60° at a scanning rate of 5 °/min. The crystallinity index (CrI) was calculated as follows.

CrI (%) = [(I_110_ − I_am_)/I_110_] × 100% (5)

where I_110_ is the maximum intensity at 2θ ≈ 20°, and I_am_ is the intensity of amorphous diffraction peaks at 2θ ≈ 16° [29].

Thermogravimetric analysis (TGA) was analyzed by a 209 F3 thermogravimetric analyzer (NETZSCH, Serbia, Germany) at a heating rate of 10 °C/min in the scope of 30 to 1000 °C under a nitrogen atmosphere. 

## 3. Results and Discussion

### 3.1. Analysis of Demineralization and Deproteinization Effect of Shrimp Shells

Shrimp shells consist mainly of chitin, protein and minerals. Chitin interacts with proteins to form chitin-protein fibers through specific hydrogen bonds [30]. The gap is full of proteins and minerals among the chitin-protein fibers [31,32]. The minerals are mainly composed of crystalline CaCO_3_. Pre-removal of minerals with citric acid helps DESs to weaken the network between chitin and proteins more easily, consequently breaking the connection within the inner structural organization of the shrimp shells. The extraction of high-purity chitin may be ascribed to the fact that DESs and shrimp shell components form hydrogen bonds, resulting in breaking the network of original hydrogen bonds in the shrimp shells. Finally, chitin is dispersed in the DESs and isolated from the proteins.

**Figure 2:**
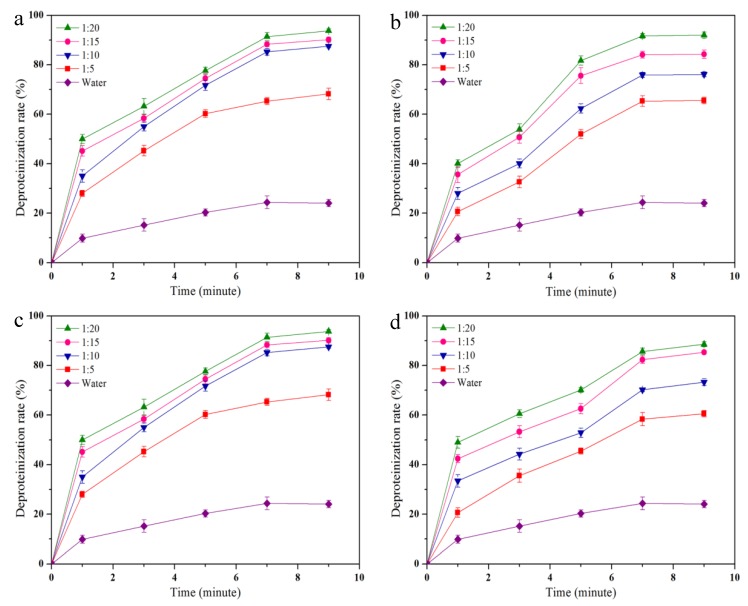
Deproteinization rates of (**a**) betaine HCl-urea, (**b**) ChCl-urea, (**c**) ChCl-ethylene glycol, and (**d**) ChCl-glycerol at shrimp shell/NADES ratios of 1:5, 1:10, 1:15, and 1:20.

The demineralization and deproteinization effect of citric acid and the DESs treatment were evaluated with different shrimp shell/DES ratios and microwave heating times. After pretreating shrimp shells with citric acid, the demineralization rate was 98.15 ± 0.3%. The deproteinization effect of DESs treatment is shown in Figure 2. The deproteinization effect increases with increasing of the shrimp shell/DES ratio from 1:5 to 1:20. This result demonstrates that the deproteinization effect could be improved at higher shrimp shell/DES ratios. Microwave heating time is also an important factor affecting the deproteinization rate. The deproteinization effect continuously improved with increasing microwave heating time, and no significant variation was observed after 7 min at all of the measured shrimp shell/DES ratios. The maximal deproteinization rates of betaine HCl-urea, ChCl-urea, ChCl-ethylene glycol, and ChCl-glycerol reached 93 ± 0.8%, 92.0 ± 1.2%, 90.6 ± 1.4% and 88.6 ± 1.1%, respectively (Figure 2a–d). The yields of chitin extracted by betaine HCl-urea, ChCl-urea, ChCl-ethylene glycol, and ChCl-glycerol were 23.6 ± 0.6%, 25.1 ± 1.3%, 24.8 ± 0.7% and 22.5 ± 1.0%, respectively, which were higher than that of the acid/alkali-extracted chitin (17.7 ± 1.8%).

### 3.2. The Degree of Acetylation (DA) of Chitin

Acetylation is an important factor for measuring the quality of chitin. The degree of acetylation (DA) of the chitin extracted by betaine HCl-urea, ChCl-urea, ChCl-ethylene glycol, and ChCl-glycerol was 92.2 ± 0.8%, 95.1 ± 1.2%, 93.4 ± 0.6%, and 91.3 ± 1.5%, respectively. However, the DA of the chitin prepared by acid/alkali method was 86.12 ± 1.4%. This result shows that DESs did less damage to the acetyl groups of chitin than strong acid and alkali. In the process of acid/alkali extraction, the intermolecular hydrogen bond of chitin was weakened significantly, which made NaOH solution easier to contact and remove the acetyl groups of the chitin, resulting in a decrease in the DA.

### 3.3. The Molecular Weight (Mw) of Chitin

The Mw of chitin is an important physicochemical property, which affects its application in various fields. The Mw of chitin extracted by betaine HCl-urea, ChCl-urea, ChCl-ethylene glycol, and ChCl-glycerol was calculated to be 3.3 × 10^5^, 3.7 × 10^5^, 3.4 × 10^5^, 2.9 × 10^5^, respectively; all of these were higher than that of the acid/alkali-extracted chitin (2.5 × 10^5^). These results suggested that the extracted chitin molecules by the DESs with the assistance of microwave were less degraded than other methods due to the relatively mild reaction conditions.

### 3.4. SEM

**Figure 3:**
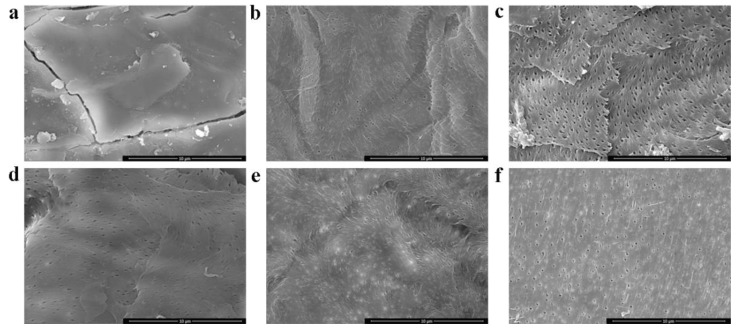
SEM images of (**a**) shrimp shells, (**b**) chitin extracted by the acid/alkali method, (**c**) chitin extracted by betaine HCl-urea, (**d**) chitin extracted by ChCl-urea, (**e**) chitin extracted by ChCl-ethylene glycol, and (**f**) chitin extracted by ChCl-glycerol.

SEM images of the shrimp shells, acid/alkali-prepared chitin, and DES-prepared chitin are presented in Figure 3. The morphology of the DES-prepared chitin (Figure 3c–f) exhibited high-density porous and fibrous structures, which were similar to those of the acid/alkali-prepared chitin (Figure 3b). On the contrary, the shrimp shells (Figure 3a) appear to have a rough surface without pores due to the presence of proteins and minerals [33]. The DES-extracted chitin show smooth surface characteristics with pores because of the elimination of proteins and minerals from the shrimp shells.

### 3.5. FT-IR

**Figure 4:**
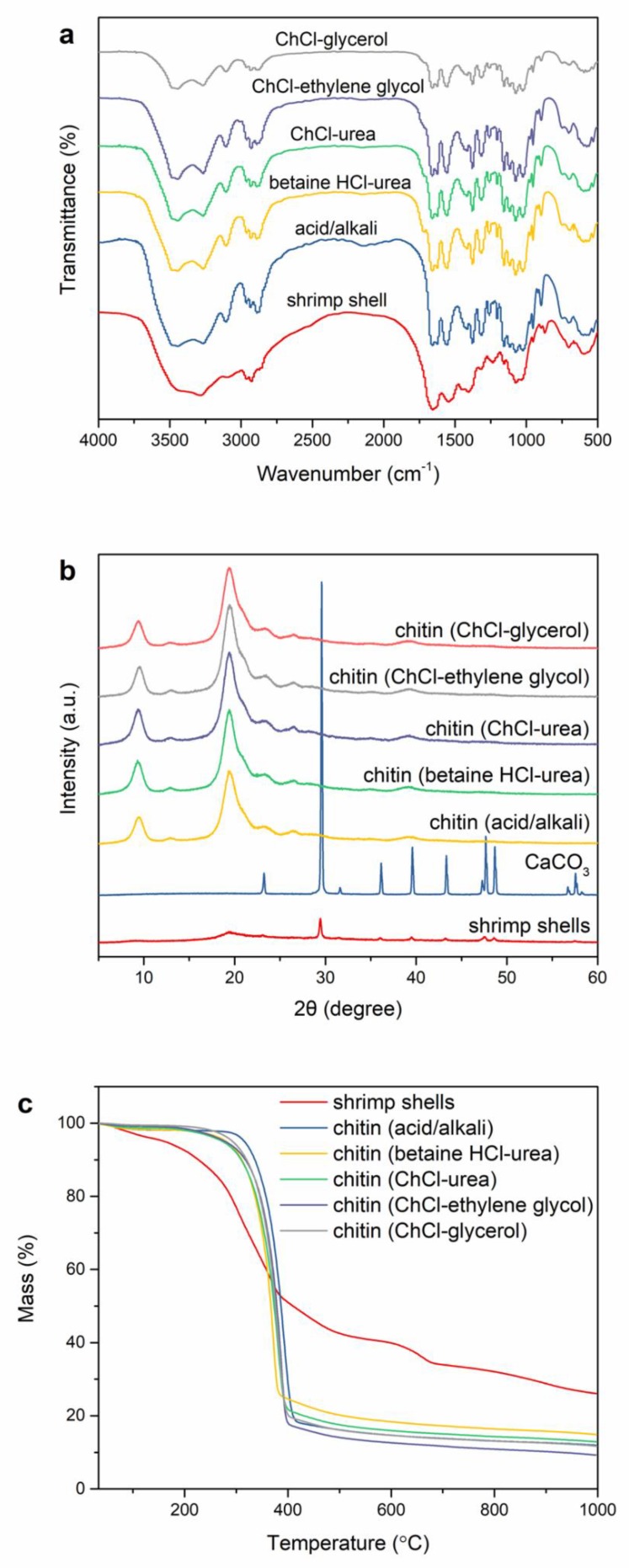
(**a**) FT-IR spectra of shrimp shells, acid/alkali-prepared chitin, and DES-prepared chitin; (**b**) XRD curves of the shrimp shells, CaCO_3_, acid/alkali-prepared chitin, and DES-prepared chitin; and (**c**) TG curves of the shrimp shells, acid/alkali-prepared chitin, and DES-prepared chitin.

The FT-IR reports of the DES-prepared chitin, acid/alkali-prepared chitin, and shrimp shells are shown in Figure 4a. The FT-IR spectra of DES-extracted chitin are consistent with those of acid/alkali-extracted chitin. The absorption peak appearing at 3449 cm^−1^ is ascribed to O–H stretching vibration (C_6_–OH…O=C) [34]. Two absorption bands at 3268 cm^−1^ and 3104 cm^−1^ are ascribed to the N–H stretching restricted by intermolecular hydrogen bond -C=O…H–N- and the NH groups of intramolecular bonding [6,35]. The amide I band divided into two absorption peaks at 1661 cm^−1^ and 1625 cm^−1^, are generated by intra-chain hydrogen bonds with NH groups (-C=O…H–N-) and inter-chain hydrogen bonds with the primary OH (-C=O-HOCH_2_-) [36]. These are typical bands of α-chitin. In addition, the absorption peaks of amide II that appear at 1560 cm^−1^ are generated by C–N stretching and amide III at 1316 cm^−1^ assigned to C–H bend [36]. For the spectrum of shrimp shell, the amide band at 1658 cm^−1^ is not clearly separated because of the overlapping of peaks of protein [37]. In contrast, an amide band was separated after DESs treatment, indicating that the proteins were eliminated from the shrimp shells.

### 3.6. XRD

To evaluate the crystal structure and crystallinity of the samples, XRD analysis was conducted on the DES-prepared chitin, acid/alkali-prepared chitin, shrimp shells and CaCO_3_ (Figure 4b). Two main diffraction peaks at 9.3°, 19.2° [38], and weak diffraction peaks at 12.9°, 23.4°, 26.4° were observed in the acid/alkali-prepared chitin and DES-prepared chitin [24,39]. These diffraction peaks are typical for the crystalline structure of α-chitin. After the DES treatment, the intensity of the characteristic peaks of a-chitin increased, whereas the peak associated with CaCO_3_ at 29.6° had disappeared in the DES-prepared chitin, suggesting that the α-chitin concentration was increased by removing CaCO_3_. Moreover, the CrI indexes of the chitin extracted by betaine HCl-urea, ChCl-urea, ChCl-ethylene glycol, and ChCl-glycerol were 70.8%, 81.0%, 80.8%, and 69.5%, respectively. The relatively low crystallinity of the chitin extracted by betaine HCl-urea and ChCl-glycerol can be attributed to the breaking of intramolecular and intermolecular hydrogen bonds and the formation of amorphous chitin [40,41]. The decrease in crystallinity of acid/alkali-extracted chitin (65.4%) is due to the swelling of chitin caused by HCl and NaOH, which makes it easier for them to enter into the chitin molecule, resulting in a larger crystal plane distance. The CrI index of the shrimp shells was found to be 48.3%, which is lower than that of the DES-extracted chitin. Overall, the increase in crystallinity indicated that calcium carbonate and proteins were eliminated from the shrimp shells through citric acid and DES treatment.

### 3.7. TGA

TGA examination of the samples was conducted to evaluate their thermal stability. Figure 4c shows TGA curves for the shrimp shells, acid/alkali-prepared chitin, and DES-prepared chitin. The initial slight decomposition before 100 °C was related to the evaporation of chemisorbed water [39]. The second stage of degradation at 100 to 250 °C was largely due to the breakdown of proteins and lipids [27]. In the third decomposition stage the samples showed large weight losses at 250–400 °C, which was attributed to the decomposition of chitin. The final weight loss in the shrimp shells between 600 and 700 °C resulted from the conversion of CaCO_3_ to CaO and CO_2_ [27]. Moreover, The TGA results of the DES-prepared chitin were consistent with those of the acid/alkali-prepared chitin, and they only showed two independent decomposition stages, namely, the evaporation of chemisorbed water and decomposition of chitin. This reveals that the chitin pretreated with citric acid was free from minerals and proteins were eliminated from shrimp shells by the DESs. 

### 3.8. Reusability

**Figure 5:**
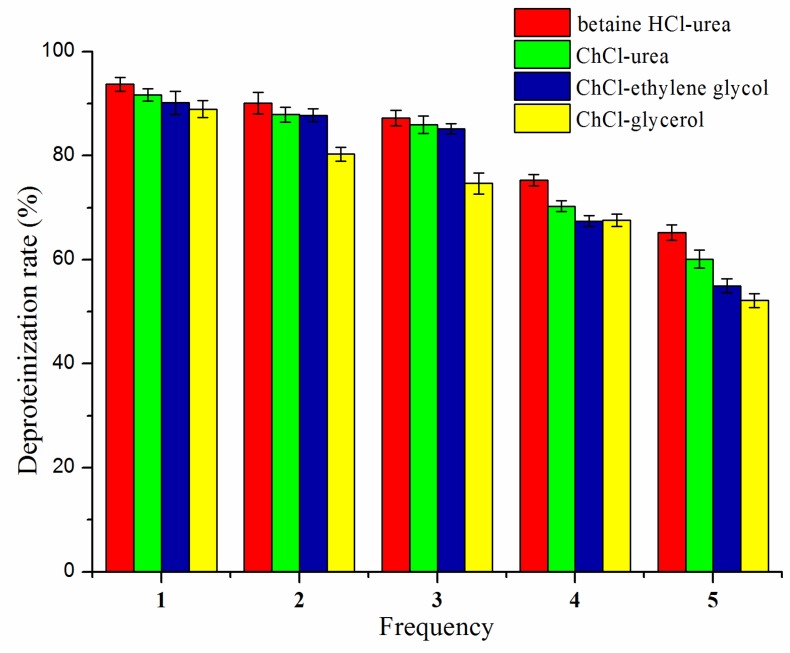
Reusability of the DESs.

As seen in Figure 5, the deproteinization rate slightly decreased after 3 cycles; however, as the number of cycles continued to increase, the deproteinization rate was significantly reduced. With the increase in reuse times, the formation of hydrogen bonds in DESs may be destroyed, resulting in poor thermal stability and low solubility, so that chitin cannot be well dissolved in DES. The betaine HCl-urea exhibited better reusability than the others. After 5 reaction cycles, the DESs became too viscous to be further reused, which might be due to the presence of proteins and other impurities in the recycled DESs.

## 4. Conclusions

In this research, a two-step extraction approach was developed and shown to be effective for chitin preparation from shrimp shells. The results showed that the demineralization rate and deproteination rate were excellent. The DA of the chitin extracted by the DESs exceeded 91%. The outcomes of SEM, FT-IR, XRD, and TGA analysis of DES-prepared chitin were similar to those of chitin prepared through the traditional acid/alkali approach, and no significant degradation of chitin occurred during the extraction process. In addition, the DESs could be reused five times. DES-based preparation avoids the utilization of harsh chemicals, which are detrimental to the environment. Taken together, this research provides an environmentally protective and effective method for chitin preparation from crustacean shells.

## Figures and Tables

**Table 1:** List of the synthesis of deep eutectic solvents (DESs) used in this study.

HBA	HBD	Molar Ratio (HBA:HBD)	Heating Temperature (°C)
Betaine HCl	Urea	1:2	50
ChCl	Urea	1:2	60
ChCl	Ethylene Glycol	1:2	60
ChCl	Glycerol	1:2	90
